# The COVID-19 Disappeared: From Traumatic to Ambiguous Loss and the Role of the Internet for the Bereaved in Italy

**DOI:** 10.3389/fpsyt.2021.620583

**Published:** 2021-05-07

**Authors:** Ines Testoni, Claudia Azzola, Noemi Tribbia, Gianmarco Biancalani, Erika Iacona, Hod Orkibi, Bracha Azoulay

**Affiliations:** ^1^Department of Philosophy, Sociology, Education and Applied Psychology (FISPPA), University of Padova, Padova, Italy; ^2^Emili Sagol Creative Arts Therapies Research Centre, Faculty of Social Welfare and Health Sciences, University of Haifa, Haifa, Israel

**Keywords:** COVID-19, traumatic loss, ambiguous loss, prolonged grief, photography, facebook

## Abstract

In Italy, in the very first phase of the COVID-19 pandemic there was a dramatic rise in mortality. However, families were forbidden because of lockdown regulations to be with their loved ones at their deathbeds or to hold funerals. This qualitative study examined bereavement experiences among family members, how they processed their grief, and how they used social networks in particular by uploading photographs during the working-through of bereavement. The sample was composed of 40 individuals aged 23–63 (80% women) from different Italian cities severely impacted by the virus, including a subgroup from the province of Bergamo, which was the city with the highest mortality rate during that time. All interviews were conducted by phone, Skype, or Zoom. Then, the transcriptions underwent a thematic analysis using Atlas.ti. The main themes that emerged were: abandonment anger and guilt, dehumanized disappeared, derealization and constant rumination, and social support and the importance of sharing photos on Facebook. Importantly, the use of social networks proved to be a valuable source of support and photographs were a powerful tool in facilitating the process of mourning by encouraging narration and sharing. Grief had a complex profile: on the one hand, it was traumatic and characterized by all the risk factors causing mourners to experience prolonged grief, but on the other, some features were similar to ambiguous loss (that occurs without closure and clear understanding) because of the impossibility to be with their relatives in their final moments. The possible relationships between ambiguous loss, the use of internet, and the risk of prolonged grief are discussed.

## Introduction

After China, Italy was the first country to suffer the dramatic and unexpected consequences of the COVID-19 pandemic (1, 2). The virus exploded in the last week of February, seriously affecting some provinces in Northern Italy, including Bergamo (3). The excess deaths (91% of the total mortality rate beyond the national average in March 2020) was concentrated in the period between February 20 (first COVID-19 deaths) and March 31. Compared to the average for the same period from 2015 to 2019, in those regions, it reached 25,354, of which 54% were the deaths diagnosed as COVID-19 (13,710) (4). In March, in the provinces most affected by the epidemic, 280% more deaths were reported (including Bergamo: 568%) (4). Local communities and health services were overwhelmed by demands for care that they were unable to handle (5). Many families saw their loved ones get into an ambulance and never return, only to receive their ashes some time later. For most, no funerals could be organized and the bereaved were forbidden to find comfort in a friendly embrace because of the lockdown restrictions.

Under such conditions, these losses can be considered traumatic (6), because they occurred in sudden and tragic circumstances. The lockdown also instituted a prolonged period of restrictions and social distancing (7), which negatively impacted people mental health (8), interfering with work and normal relationships of caring and social support (9), including end-of-life rites and funeral rituals. For this reason, the loss resulted in a traumatic experience, especially because it was impossible to take leave of even the most important attachment figures.

Some of the bereaved spontaneously gathered in virtual groups on Facebook during the lockdown, where they could talk about this painful experience and deal with the unusual death of their loved ones. A virtual community of self-help and mutual aid groups emerged in which participants could express their grief over their loss by posting their thoughts or photographs of their deceased loved ones. The internet became a psychological living space that responded to needs that could not be satisfied in the real world, and allowed individuals to create a dimension where their mourning could be socially shared (10). The term “thanatechnology” refers to the computer tools through which information about deceased persons, forms of commemoration, and forms of psychological and spiritual support can be provided via the internet (11, 12). Research on grief and mourning in digital environments often focuses on the cultural practices and meanings that are played out through digital means (12). Thus, the internet has modified the approach to loss and death, because the image of the deceased can be seen by anyone who accesses the site anywhere in the world. In particular, researchers have described how in many cases, relative and friends can rekindle the memory of the dead by uploading photos on the deceased's personal page (12). This practice seems to be an important source of social support for mourners, who draw strength from the comments and the messages shared within the community (13). The sharing of photos and videos made it possible to involve other individuals and gain visibility, thus attesting to the existence of the presence of the deceased (14). This strategy takes the form of remembering and being remembered (15). Studies have underscored the importance of the use of photographs in the management of grief and mourning, both in real life (16, 17) and on the internet (18).

In Northern Italy, in the areas most affected by the pandemic in the first phase, a virtual community was formed. Its purpose was to offer participants a space for the commemoration of their dead and mutual support. In the present study, we discuss the results of a qualitative investigation of this experience and the elaboration of traumatic grief through the internet by some of mourners who lost their loved ones to COVID-19.

The aim of this qualitative study was to investigate whether and how bereaved individuals coped with their mourning during the lockdown and in its aftermath through the use of Facebook. In particular, we investigated how the photographs uploaded to social networks were used, and how they were shared. We also explored the characteristics of this kind of mourning. We hypothesized that this type of mourning would presents specific characteristics that could best be explored by a grounded (i.e., bottom-up) perspective.

## Materials and Methods

### Participants

The sample was composed of 40 individuals aged 23 = 63 (*M* = 47, *SD* = 9.85; 80% female) from different Italian cities hard hit by COVID-19. A subgroup (13 out of 40) lived in the province of Bergamo, one of the areas most severely affected by the virus (3, 19). Three of the participants lived outside Italy but their deceased family member lived in Bergamo. Participants were recruited using a convenience sampling. This non-probabilistic strategy does not allow to generalize the results. Participants were contacted by researchers through a Facebook group founded by those who had lost loved ones because of COVID-19 and expressed their grief by sharing their stories and photographs of their deceased on the forum. The number of participants stopped at 40 as the topics were saturated. The number of participants is appropriate for a qualitative research, according with the standards required for qualitative method (20, 21).

### Data Collection and Data Analysis

Each participant was interviewed by phone, Skype, or Zoom, with an average of 60 min for call. The interview method was explained in advance to the participants, so they were asked to choose which channel was most convenient for them. Participants who were less skilled with technological devices opted for a phone call, those who were more skilled opted for the use of Skype or Zoom platforms. A semi-structured interview, specifically created for the collection of research data, was conducted. Specifically, it consists of 12 questions divided into four thematic areas: the narration of the experience (four questions), perceived resources or misses (three questions), funeral rituals (three questions), and personal beliefs about the themes of death (two questions) (see Appendix A). The interview aimed to explore the resources the participants drew upon to deal with their pain and the lack of physical support from friends and relatives, when dealing with the trauma of not being able to be physically with or say goodbye to their loved one. The main topics addressed in the interviews were the experiences related to the painful event before and after the news of the death of their relative and the resources they tapped to deal with the event and the mourning process. Particularly, participants were asked how Facebook had been experienced, and were asked to show the interviewer the photos uploaded to the website, to facilitate dialogue and memories. The study was inspired to the Grounded Theory perspective and involved qualitative research in psychology with in-depth interviews, because this approach makes it easier to understand subjective stories (22, 23). The Grounded Theory offers a practical and flexible approach to interpret complex social phenomena not yet sufficiently studied. Indeed this approach does not start with testing an existing hypothesis but uses the empirical data to generate concepts and theories (22, 23). Thus, one of the benefits of this perspective is the actively reflective position of the researcher while interacting with data, which involves the generation of analytical categories and the identification of relationships between them. The thematic analysis method was adopted to analyze the data, because it offers an accessible and theoretically flexible approach, and it is consistent with the framework of the Grounded Theory (24). Indeed, the conversations were recorded and transcribed in preparation for the Thematic Analysis, in which we identified the patterns of themes using a bottom-up approach (8, 25). All the texts were processed using Atlas.ti software (26), which allows researchers to work directly on written texts, highlight portions of them, create labels to be inserted in each text that can adequately represent the fundamental themes, and construct larger clusters of meaning by comparing the data obtained from each text (27). The numbers indicated in each quote in the following description of the results specify the interview number that corresponds to each participant (from 1 to 40) and the order number refers to the place of quote in the working text in Atlas.ti. Four researchers who conducted this study offered their expertise in qualitative data analysis and code identification. The research followed APA Ethical Principles of Psychologists and Code of Conduct and the principles of the Declaration of Helsinki, so participants were explained in detail all the objectives of the research and the methodology of analysis used. They were asked permission to record the conversations, to transcribe their answers and to analyze their contents in order to study the phenomenon. We have guaranteed them to anonymize the contents of the obtained texts and only those who have given written and signed consent have participated in the research. This study was approved by the Ethics Committee for Experimentation of the University of Padua (n. 27331296116C7206D8A5B61A06F4845B). In addition, a written informed consent was obtained from the author of the photographs to freely use the photographs for the publication of this article.

## Results

Most of the participant of this study had lost their father (22 people out of 40) or mother (16 people out of 40); six participants had two losses: five had lost both parents and one had lost his father and grandfather; three of the respondents had lost one of their grandparents, three wives had lost their husband, and finally two participants out of 40 had lost their brother. With respect to level of education, 12 subjects of the participants had an academic degree (including PhD), 16 out of 40 had a junior high school level, and the others (12 out of 40) had high school diplomas. All believers were Christian, except one who was a Buddhist. Specifically, 19 participants out of 40 stated they were believers but not practitioners (among whom seven of them who had lost their faith because of COVID-19); 12 subjects out of 40 were believer and practitioners (among whom five of them had lost their faith, whereas three of them had strengthened their faith in God); 6 people out of 40 were atheists and one person did not respond.

The analysis yielded the following four main themes: abandonment anger and guilt, dehumanized disappeared, derealization and constant rumination, and social support and the importance of sharing photos on Facebook (Table 1). Illustrating photos are in the online Supplementary Material File. All the names cited below are fictitious (pseudonym) to prevent any possibility of identification of the participants.

**Table 1:** Results.

**Thematic area**	**Code**	**Frequency**	**Example**
1. Abandonment: anger and guilt	(a) Feeling abandonment by the health services	34	The healthcare system failed to respect the right to care of its citizens. It did not respect the right of every citizen to be treated. My father told me on the last day that I spoke to him that “we have been abandoned,” and I think this is true, not because of the doctors but because of those above them who were unable to handle the emergency. The physicians and nurses are not guilty because it was a terrible experience for them too, and many of them died because they didn't even know what was happening. Both my parents fell ill and were not protected.
	(b) Desire of revenge	17	At that moment I was not thinking about the lack of family but about my mother, who had passed with no one near her to watch over her and certainly did not deserve to die in that inhuman way. They covered her with a sheet and zipped up a black body bag. That's all. It is not the right way to treat a human being who dies in this way.
	(c) Sense of abandonment their loved	19	First of all, what makes us all suffer is the fact that we could not be near her, holding her hand in her last moments. I feel stupid saying this, but I suffer because I feel I abandoned my mother at the most important moment and inside, I imagine her dying alone. When I was finally able to go to her grave, I apologized to her and explained that I was not allowed to be near her when she died.
2. Dehumanized disappeared	(a) Non-acceptance of reality	30	What doesn't allow me to accept reality, like all those who have had an experience similar to mine, is the fact that I abandoned my mother. My mother in this way has become just a number in the daily statistics on the lethality of the virus. I could not see her again. It is as if she had disappeared.
	(b) Lack of humanity	16	It is incredible, but I could not be next to my father during his illness, he died alone, even afterwards I had no opportunity to say goodbye to him. He was treated like a piece of dead meat, he was taken naked, without clothes, they washed him down with disinfectant and he was put in a black bag. It was heart-breaking, these are all things that give me no peace and make me think that my father was just a piece of meat.
	(c) Lack of funeral ceremonies	17	I can't get over what happened. Not being able to hold a funeral for my brother made everything difficult for me. A funeral, whether religious or not, is a moment that unites the bereaved who gather and process the sense of loss and take stock of the reality of the end.
	(d) Derealization	30	Like others with whom I share experiences on Facebook, it often seems like a dream to me too. I still don't really realize what happened. I know he died, rationally, but I can't close the circle without having seen him and without being able to accompany him. It seems to me that everything could go back to the way it was before. He is going to come through the door and that I can wake up from this nightmare.
3. Derealization and constant rumination	(a) Need for clarity	13	It is all too unclear. It is not clear what happened. I'm waiting for the medical records to see, to be able to make sense of it and give me concrete explanations.
	(b) Constant rumination	23	At first I found it hard to believe it because it seemed impossible that it could happen like this. So I started brooding, saying “But no, it's not possible, it can't be like this, what went wrong? It can't have been like that.” And the more I think about it, the angrier I get, it's really difficult to manage because the pain of not being able to see my father for 5 weeks, and in the end not being able to be with him when he died is still unbearable and I keep thinking about it.
	(c) Insomnia	14	We are all afraid of getting sick, of infecting others and afraid that everything, when we get back to normal, will be forgotten. We were up all night waiting for calls from the hospital. Even now it is difficult to fall asleep.
4. Social support and the importance of sharing photos on Facebook	(a) - Religion source of support - Religion as an impediment	7	The dialogue with God helped me. Religion supported me. At times like this, you either lose faith or gain faith.
		6	Anger makes me think that my father is now just dust. What God can allow all this? What plan does God have for all this to happen? By now I believe that if a God exists, He is a Nazi. Tonight there will be a mass for my father, but right now I have to say.what do I care?
	(b) Support from Social Network	29	I liked sharing this photo because here my mother was lively, she smiled, she could move. It was her wedding anniversary, we had prepared a small cocktail party and she was happy, my mother loved to eat, she ate to sample and enjoy. Remembering her like that gave me relief and helps me to think of her as happy.
	(c) Importance of sharing photos on Facebook	15	I tried to show the anguish through photography. This angel looks out the window. An angel should be in heaven, instead he stands here, looking out the window. I mean we are the angels who are on Earth and the angels who are in heaven are the stars that illuminate us at night.

### Theme 1: Abandonment: Anger and Guilt

#### Feeling Abandonment by the Health Services

All the participants experienced an intense feeling of abandonment by the health services who were unable to provide care in this extraordinary emergency crisis. For example, Amelia talked about here experience: “The healthcare system failed to respect the right to care of its citizens. It did not respect the right of every citizen to be treated. My father told me on the last day that I spoke to him that ‘we have been abandoned,' and I think this is true, not because of the doctors but because of those above them who were unable to handle the emergency. The physicians and nurses are not guilty because it was a terrible experience for them too, and many of them died because they didn't even know what was happening. Both my parents fell ill and were not protected” [7:13].

#### Desire of Revenge

Resentment also characterized all the narratives, accompanied by a desire for revenge that led the participants to join to a committee formed to condemn the situation and take legal action against the health care system. The most outrageous was the impossibility to say goodbye to their relatives and not being able to hold a funeral. For example, Clara stated “At that moment I was not thinking about the lack of family but about my mother, who had passed with no one near her to watch over her and certainly did not deserve to die in that inhuman way. They covered her with a sheet and zipped up a black body bag. That's all. It is not the right way to treat a human being who dies in this way” [2:19].

#### Sense of Abandonment Their Loved

The lockdown exacerbated this experience of anger and resentment, loading it further with a strong sense of powerlessness and helplessness caused by the impression of having abandoned loved ones. Cinzia stated “First of all, what makes us all suffer is the fact that we could not be near her, holding her hand in her last moments. I feel stupid saying this, but I suffer because I feel I abandoned my mother at the most important moment and inside, I imagine her dying alone. When I was finally able to go to her grave, I apologized to her and explained that I was not allowed to be near her when she died” [21:19].

### Theme 2: Dehumanized Disappeared

#### Non-acceptance of Reality

This anger and discouragement was accompanied by a non-acceptance of reality because it was unacceptable to have abandoned loved ones to their fate without being able to see them again. Speaking about his mother who died in a nursing home, Mauro said “What doesn't allow me to accept reality, like all those who have had an experience similar to mine, is the fact that I abandoned my mother. My mother in this way has become just a number in the daily statistics on the lethality of the virus. I could not see her again. It is as if she had disappeared” [11:2].

#### Lack of Humanity

The idea of dehumanization characterized the sense of unreality of the experience, as Ludovica said: “It is incredible, but I could not be next to my father during his illness, he died alone, even afterwards I had no opportunity to say goodbye to him. He was treated like a piece of dead meat, he was taken naked, without clothes, they washed him down with disinfectant and he was put in a black bag. It was heart-breaking, these are all things that give me no peace and make me think that my father was just a piece of meat” [10:4]. Luca discussed the dehumanization of pandemic victims, saying “Even when the numbers are high, they are just numbers. It is not detectable physically, it is ethereal and different from a devastating earthquake where there is solidarity and is palpable, like the tsunami in Indochina where everyone donated a euro to support them. Here no one feels any solidarity. Facebook and the sharing of stories and photos are a form of recognition, a restoring of a human identity to the dead, because no one has given it to them” [11:12].

Edoardo referred to the fact that his city commemorated the victims of COVID-19 with an installation of objects which have a symbolic meaning and commented: “The idea is good, but if we do not put first and last names and photographs on those objects, people are denied once again. People who died had a first name and a last name, so we have to put some history, otherwise this is not a way to remember that they were people and not just numbers.”

#### Lack of Funeral Ceremonies

The further lack of funeral ceremonies did not allow the bereaved to process the loss in a healthy way, as Asia pointed out: “I can't get over what happened. Not being able to hold a funeral for my brother made everything difficult for me. A funeral, whether religious or not, is a moment that unites the bereaved who gather and process the sense of loss and take stock of the reality of the end” [16:23].

#### Derealization

The impossibility of accompanying one's loved one on their final journey triggered a strong feeling of derealization (i.e., feeling detached from the external world that seems distorted and unreal), an incredulity that was characterized by brooding over negative thoughts, as expressed clearly by Ilaria: “I still can't realize that my mother is dead. I keep thinking about all the possible scenarios of her death… If I had kept her at home maybe she would still be alive. Or if they treated her like an animal. She couldn't breathe and they gave her morphine to make her die. All the dead in this country died in this way, like animals” [19:8]. Denise said: “Until recently, I still believed she was still in the nursing home. My grandmother had underlying conditions but she was fine and …. not having seen it with my own eyes when she died I find it hard to think that she is not there and that I cannot go to see her” [1:7]. Adelaide also described a similar experience “Like others with whom I share experiences on Facebook, it often seems like a dream to me too. I still don't really realize what happened. I know he died, rationally, but I can't close the circle without having seen him and without being able to accompany him. It seems to me that everything could go back to the way it was before. He is going to come through the door and that I can wake up from this nightmare” [15:2].

### Theme 3: Derealization and Constant Rumination

#### Need for Clarity

Federica still felt unable to acknowledge that her beloved was dead because she was convinced that there were too many inconsistencies and for this reason she suspects that there were mistakes that have been concealed to the public: “It is all too unclear. It is not clear what happened. I'm waiting for the medical records to see, to be able to make sense of it and give me concrete explanations” [2:20].

#### Constant Rumination

The search for evidence triggered constant rumination in almost all participants, as Emily described: “At first I found it hard to believe it because it seemed impossible that it could happen like this. So I started brooding, saying ‘But no, it's not possible, it can't be like this, what went wrong? It can't have been like that.' And the more I think about it, the angrier I get, it's really difficult to manage because the pain of not being able to see my father for 5 weeks, and in the end not being able to be with him when he died is still unbearable and I keep thinking about it” [13:5].

#### Insomnia

Another typical feeling that led to insomnia was fear, as described by Margherita: “we are all afraid of getting sick, of infecting others and afraid that everything, when we get back to normal, will be forgotten. We were up all night waiting for calls from the hospital. Even now it is difficult to fall asleep” [30:31]. Ruminating causes insomnia, as Adriano, said: “I don't sleep much, I wake up in the middle of the night and my thoughts go straight to my mother because I don't know how she spent those 4–5 days in the hospital before she died. She disappeared and I don't know where she went, what happened to her, how she died. I don't sleep anymore and I think about it, I wonder if she was lucid or if they sedated her with some medication. Then I think about my brother and when he was able to see her. At that time, she took off her watch and gave it to him. She never took off her watch. It was a message ‘take my time because mine is over.' I keep thinking back to when they loaded her into the ambulance and she disappeared. That was the last time I saw her. I can no longer sleep and I keep thinking about all this” [8:16]. He continues: “I live this loss probably like the relatives of the 85 who died in the [1980] Ustica plane that crashed. Even 40 years later, they still don't know what happened and have not received an explanation” [8:24].

### Theme 4: Social Support and the Importance of Sharing Photos on Facebook

Analysis of the participants' descriptions of their sources of support to manage their loss highlighted the importance of close relatives and friends, and sometimes neighbors. Funeral home directors emerged as some of the most pro-social figures who were able to support the bereaved during the funerals and the cremations. Some also found comfort in work, hobbies and volunteering that allowed them to overcome their sense of helplessness.

#### Religion

Religion was not considered an unequivocal source of support. Some found sustenance in God like Emiliano: “The dialogue with God helped me. Religion supported me. At times like this, you either lose faith or gain faith” [33:6]. Michele said: “Anger makes me think that my father is now just dust. What God can allow all this? What plan does God have for all this to happen? By now I believe that if a God exists, He is a Nazi. Tonight there will be a mass for my father, but right now I have to say.what do I care?” [6:15]

#### Support From Social Network

The most significant source of help, however, was found on the internet. Most of the participants stated that they found real support especially in the Facebook group dedicated to the COVID-19 grievers, where the participants can upload a photo of the deceased. Matilde said: “In the Facebook group, I felt held in a hug of tears, some like my own, some worse than my own who told me they had lost both their parents. I was happy because sometimes sharing tears makes you feel like you are in an embrace even if they were just tears” [21:15]. Crystal said: “I think that only those who have gone through an experience like mine can understand me. People who have not experienced something like this cannot understand. In the Facebook group I could post my father's photo because I knew that the others were in a very similar condition and were having the same feelings” [3:17]. Rebecca expressed these ideas in a similar way: “That group really made me feel like I belonged to a group of people who had been through the same drama as me and would understand me. We were all united because they had suffered mourning in the same way. That's why interacting with them made me feel good. We wrote to each other that we loved each other even though we didn't know each other and this made me feel good” [22:17].

#### Importance of Sharing Photos on Facebook

Overall, at the time of the interviews, the participants had posted a total of 292 photographs. Most of them portrayed the deceased and their distinct personalities. During the interview, the participants also produced other photos that depicted family life in moments of happiness and special occasions. Rossana talked about a photo taken when her beloved one was still feeling well: “I liked sharing this photo because here my mother was lively, she smiled, she could move. It was her wedding anniversary, we had prepared a small cocktail party and she was happy, my mother loved to eat, she ate to sample and enjoy. Remembering her like that gave me relief and helps me to think of her as happy” [40:16]. Angelica said: “These dead have only been counted, their names and their lives have not been remembered by anyone. Thanks to sharing the photos in this group, we were able to give a face to them and talk about them” [10:14]. Gina told us: “This trauma has strengthened family ties. Now we see each other more often and we go to my parents' house on Sundays for lunch. We keep these photographs, so we feel they are looking at us, as if our parents were there. But then, when we close the door in the evening, we suffer intensely because it is all over and they are not there” [17:20]. Jonathan explained how photography helped him through this painful juncture. He shared the photos of a project he did for a local magazine that published his photographs on the pandemic, and stated: “I tried to show the anguish through photography. This angel looks out the window. An angel should be in heaven, instead he stands here, looking out the window. I mean we are the angels who are on Earth and the angels who are in heaven are the stars that illuminate us at night” [29:19].

## Discussion

The COVID-19 pandemic, with its limitations, forced many people to have difficulty accessing mental health services, at a time when they would be most needed (11). In particular, the loss of a loved one during the pandemic proved to be a strong stressor in more Countries (28). The official data indicate that in the first phase of the COVID-19 pandemic there were massive numbers of sudden deaths (29). The rarity of this type of event, combined with the lack of social support (7) contributed to increasing the levels of suffering caused by the loss. The analysis of our interviews showed that the grief caused by the first emergency phase of the pandemic can be considered traumatic (30). This sudden and tragic loss led to an existential crisis because the participants' ability to adapt to the new reality and to accept what happened was abnormal and profoundly altered by the pandemic. All the narratives presented typical traumatic traits reflecting shock, anger, frustration, guilt, fear, helplessness, and anxiety. It appears that the participants experienced severe post-traumatic stress (31, 32); namely, rumination, recurring nightmares and insomnia, weight loss, loss of confidence in society, derealization. Several factors exacerbated the experience of loss. Consistent with the literature, this study also suggests that the traumatic characteristics of grief experienced during the pandemic included the multiple deaths that led to an “overload of mourning” that undermined family resilience, the high level of contagiousness of the virus that prevented the mourners from assisting their loved ones during the last days of their lives (generating a sense of helplessness and guilt), the absence of funeral ceremonies and/or in some cases the obligation of cremation in which people found themselves in a conflict between the desires of the deceased and the societal dictates (33).

In addition, the communication of bad news took on exemplary importance. Almost all the participants reported that they were told of the death of their relatives coldly. Being informed of the death of a loved one is a significant moment that can change the life of those who will suffer the loss forever. The terms used to inform the survivors and the nature of the person who provides this information influence the way survivors cope with one of the most difficult moments in their lives (34). Similarly, the perception of dehumanization of their relatives, who were depicted as numbers, or “dead flesh” without a biography, further exacerbated the trauma. Dehumanization is closely linked to de-legitimization. The literature has noted how excessive and collective accumulation of deaths deny the acknowledgment of each individual's bereavement (33).

Studies have suggested that the emotional experiences characterizing the grief experienced during the pandemic (suffering, anger, fear, guilt, remorse, and helplessness) could cause prolonged grief disorders (35). The inability to say goodbye to the deceased (36), excessive guilt (37), and a lack of social support (38) may prevent closure and cause prolonged grief.

This form of prolonged grief may resemble ambiguous loss that occurs without closure or clear understanding. Some studies have in fact described on the one hand how important it is to have direct contact with the corpse for the closure of mourning, because the contact with the corpse and funeral rituals facilitate the work of mourning (39). By contrast, the imposed lack of contact with the corpse of the deceased may lead to derealization and estrangement that impedes the mourning process (40). Thus, the mourning may be prolonged because of the ambiguity generated by a situation of “unreality.” The participants' continuous rumination, which oscillated between anger and disbelief, shows that there was a lack of a concrete foothold to which to attach the ritual scenario of loss and farewell. The internet emerged as a substitute for ritual processing made impossible in the real community by the lockdown. Literature on users of social media shows that thanatechnology permits the death of a loved one to be a moment where the deceased can acquire a lasting, infinitely replicable presence (12). As showed by Hieftje (41), posting pictures on social networks such as Facebook may create a sense of belonging and community during the bereavement, while providing continuous connections between the living and the dead. Specifically, photos help create virtual memorials, which help mourners shift death and grief from private to more public experiences. These practices are aimed to create a digital immortality, or a form of survival of the deceased in society (42, 43), because digital data stored online reflect the personality and memories of the deceased and represent the idea of a posthumous maintenance of a personhood that transcends the boundaries of the body (44). In this study, this strategy emerged as particularly useful and supportive since it allowed the participants to feel less lonely, to embrace each other at a distance that was experienced as not so remote. On the one hand, as mentioned in the literature (45), the use of Facebook proved to be fundamental to give the bereaved a space for commemoration by restoring a humanity to their relatives and enabling them to feel understood in their pain and dismay by those who had lived through the same experience. Furthermore, the internet enabled the participants to feel part of a community that alleviated the loneliness experienced during the lockdown. The evocative properties of photography proved particularly useful in creating a common ground between the interviewer and interviewees. Initially they were asked to explain why they decided to post pictures on the Facebook group, and then were asked to show other significant photographs that would tell something about the deceased, which enabled them to grapple with the “suspended” generated by such a sudden and traumatic loss. It was an opportunity to say one last word, to express what they would have liked to say to their loved one. Photography facilitated the dialogue with the interviewers, as it did on Facebook. The use of photography is known to support dialogue even around painful issues (46). The fact that the photographs were posted on Facebook and shared with the interviewer generated a sort of acknowledgment of the COVID-19 victims who were given a face, a name and a story. On the other hand, however, sharing one's experience on social networks may create a sense of immortality for the deceased, strengthening the social relationships that one has with the deceased (13), instead of placing the loved one in a symbolic dimension (47). Other creative approaches, including arts therapies and psychodrama (48–53), are likely to be helpful in processing taxing emotional experiences of trauma, loss and bereavement.

## Conclusion

This study lends weight to the claim that the grief caused by COVID-19 can be considered both traumatic and de-legitimized, and in this sense comparable to the effects of ambiguous loss. All these characteristics are linked to the experiences of dehumanization of the deceased, the lack of contact with the dying relative and the corpse of the deceased, and the absence of funeral rituals. In this scenario, for our participants, the use of social networks proved to be a significant source of personal support, which enabled them to restore an identity to the deceased. The Facebook group created a space for sharing in which they could express their experiences and feel understood. Photography has proved to be a useful tool to facilitate the elaboration of grief, encouraging a process of narration and sharing that re-equilibrated the relationship with the deceased and helped reflect on the loss, and also proved to be a vehicle of recognition of the deceased. However, numerous factors described in the literature lead us to suspect that these people could develop a form of grief without closure, because of the use of the internet, although it is still too early to come to a conclusion. In fact, the main limitation of the study is the absence of a follow-up that would allow us to verify the condition of the participants with respect to their use of the internet. Furthermore, one of the major limitations of the research is the non-generalizability of the results for three main reasons: the first one is that the group of participants is too small; the second one is that the group of participants consists mostly of sons/daughters who have lost their parents; the last but not the least is that qualitative studies are not aimed to obtain generalizable results. In particular research inspired to he Grounded Theory approach highlights possible scenarios that could be further investigated in a second time, utilizing different methodologies. In this case, it would be useful to investigate the possible presence of continuing bonds and their effect on grief counseling. These could constitute the groundwork for future research with the same participants.

## Data Availability Statement

The original contributions presented in the study are included in the article/Supplementary Material, further inquiries can be directed to the corresponding author/s.

## Ethics Statement

This study was approved by the Ethics Committee for Experimentation of the University of Padua. The patients/participants provided their written informed consent to participate in this study.

## Author Contributions

IT is the scientific director of this research project. She designed the study and supervised its implementation. She also contributed to the data analysis the interpretation of the results, supervised the preparation of the manuscript, and wrote its final version. CA and NT were the researchers directly responsible for the data collection and its analysis. GB and EI supervised the data collection and analysis. HO contributed to the writing of this article. BA participated in discussions on the results. All authors contributed to the article and approved the submitted version.

## Conflict of Interest

The authors declare that the research was conducted in the absence of any commercial or financial relationships that could be construed as a potential conflict of interest.

## Supplementary Material

Figure S1 This is my little prince looking at, or rather suffering for, the Earth.

Figure S2 This angel is looking at the sky out of the window, instead of where an angel should be, in the heavens. However, the difference is that we are angels on earth and the others, who are above, are the stars that light up the night.

Figure S3-S5 Tonight I want to share with you some pictures that are very important to me, somehow concerning one of the situations exasperated by COVID-19: the silence inside homes. Sexual abuse and violence against women who right now cannot find any relief. Do not remain alone; love exists. Do not hesitate to contact the number 1522 (free and active 24 hours a day). Let's help change the world to make it better! True love is there. He managed to bend you; he managed to mortify your body until you got down on your knees and made you believe you had to hide. He, full of himself, tries to look at his image in that mirror, but the mirror refuses to reflect that horror. He dragged you into the darkness because your light scares him. Your light, so pure and strong, forces him to recognize the darkness he has inside. Turn around, get up. Feel and taste your light again. No one is as bright as someone who trembles in the dark.

Figure S6 Our Presolana is crying, the Serio river carry the tears of the Valley . I want to dedicate to all those People who have left us and now, from up there, close to the stars, will illuminate our sky and our dreams . They are the people that have contributed to the growth of life, who have made the history of our countries. Never shall we forget them, for everyone has a story to tell.

Figure S7 This represents the image of this person with an umbrella, going for a journey.

## Appendix A. Interview

**1. Narration of the Experience**

a May I ask you to tell me about your experiences before and after the news of the decease?
b Tell me what it means to you to have published this photograph; in particular, what made you choose this one instead of another?
c May I ask where and by whom this photograph was taken? In your opinion, what aspect(s) of your loved one does this photograph represent?
d Are there any photos other than this one that you feel are particularly significant, the most beautiful ones?

**2. Perceived Resources or Misses**

a Can I ask you to tell me, during the lockdown period, what/who you missed in order to cope with this grieving situation? Is there something/someone you were able to find some support from instead?
b Which tools did you find useful to share your experience? Have you used the psychological support numbers made available by institutions?
c How did posting this photograph on Facebook make you feel? Was there any feedback from other participants? If so, what kind?

**3. Funeral Rituals**

a How did you experience the lack of funeral ritual? How was the body handled?
b How do you feel about cremation? (and if the deceased loved one's body was cremated) Would you have chosen it as a form of funeral rite at a time other than COVID-19? Has your opinion about cremation changed during this period?
c To date, having been able to participate in a religious ritual again, what has it made you feel?

**4. Personal Beliefs about the Themes of Death**

a If your deceased loved one were here, if you could imagine yourself standing next to him/her right now (use the photograph if necessary) what do you think he/she would say? What would you say to him/her?
b According with what you expressed in the biographical form, you told me regarding beliefs that you believe in the afterlife/you do not believe in the afterlife; can I ask you where you imagine your deceased loved one is right now if you think about him/her?
