# Supplementary figures and images for: The COVID-19 Disappeared: From Traumatic to Ambiguous Loss and the Role of the Internet for the Bereaved in Italy
Source: Front Psychiatry. 2021 May 7;12:620583. doi: 10.3389/fpsyt.2021.620583 (PMC8138554; doi:10.3389/fpsyt.2021.620583)

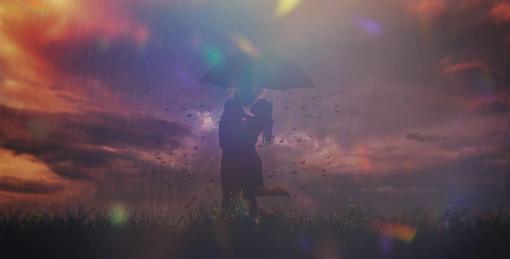

Supplement: Figure S1 — This is my little prince looking at, or rather suffering for, the Earth. [file Image_1.jpeg]

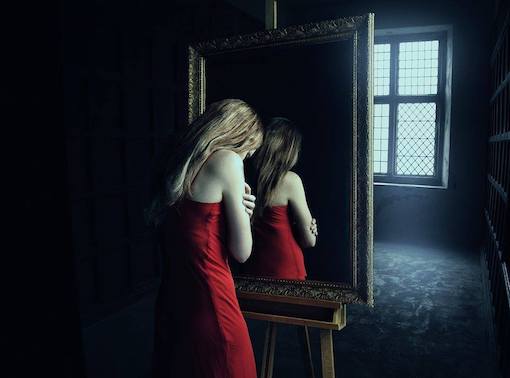

Supplement: Figure S2 — This angel is looking at the sky out of the window, instead of where an angel should be, in the heavens. However, the difference is that we are angels on earth and the others, who are above, are the stars that light up the night. [file Image_2.jpeg]

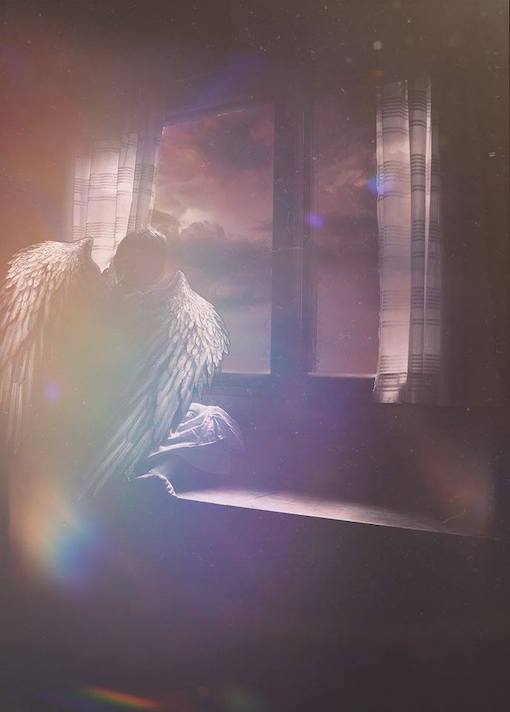

Supplement: Figure S3-S5 — Tonight I want to share with you some pictures that are very important to me, somehow concerning one of the situations exasperated by COVID-19: the silence inside homes. Sexual abuse and violence against women who right now cannot find any relief. Do not remain alone; love exists. Do not hesitate to contact the number 1522 (free and active 24 hours a day). Let's help change the world to make it better! True love is there. He managed to bend you; he managed to mortify your body until you got down on your knees and made you believe you had to hide. He, full of himself, tries to look at his image in that mirror, but the mirror refuses to reflect that horror. He dragged you into the darkness because your light scares him. Your light, so pure and strong, forces him to recognize the darkness he has inside. Turn around, get up. Feel and taste your light again. No one is as bright as someone who trembles in the dark. [file Image_3.jpeg]

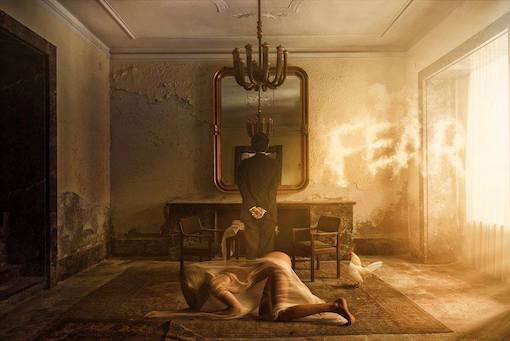

Supplement: Supplementary file 4 [file Image_4.jpeg]

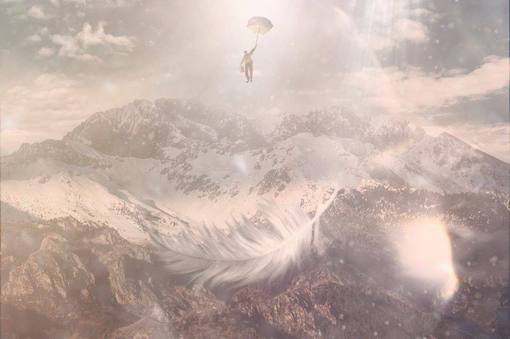

Supplement: Supplementary file 5 [file Image_5.jpeg]

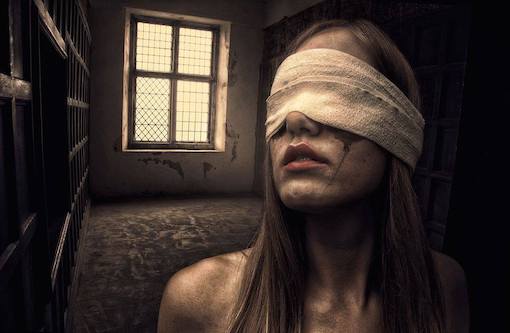

Supplement: Figure S6 — Our Presolana is crying, the Serio river carry the tears of the Valley . I want to dedicate to all those People who have left us and now, from up there, close to the stars, will illuminate our sky and our dreams . They are the people that have contributed to the growth of life, who have made the history of our countries. Never shall we forget them, for everyone has a story to tell. [file Image_6.jpeg]

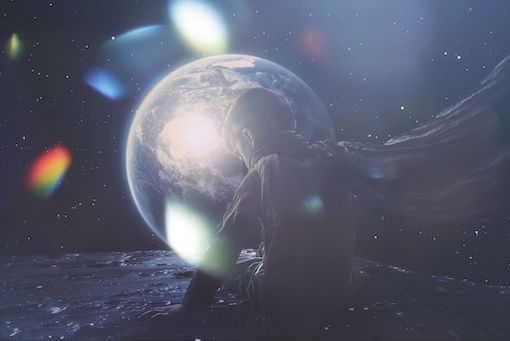

Supplement: Figure S7 — This represents the image of this person with an umbrella, going for a journey. [file Image_7.jpeg]
